# Intelligent Recommendation Systems Powered by Consensus Neural Networks: The Ultimate Solution for Finding Suitable Chiral Chromatographic Systems?

**DOI:** 10.1021/acs.analchem.4c02656

**Published:** 2024-07-10

**Authors:** Salvador Sagrado, Carlos Pardo-Cortina, Laura Escuder-Gilabert, María José Medina-Hernández, Yolanda Martín-Biosca

**Affiliations:** † Departamento de Química Analítica, Universitat de València, Burjassot, E- 46100 Valencia, Spain; ‡ Instituto Interuniversitario de Investigación de Reconocimiento Molecular y Desarrollo Tecnológico (IDM), Universitat Politècnica de València, Universitat de València, E-46100 Valencia, Spain

## Abstract

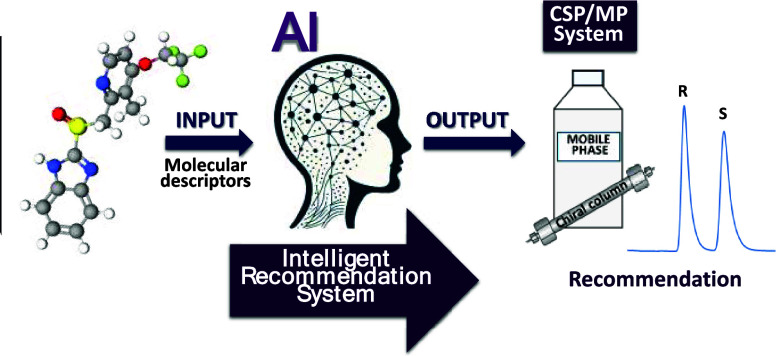

The selection of suitable combinations of chiral stationary phases (CSPs) and mobile phases (MPs) for the enantioresolution of chiral compounds is a complex issue that often requires considerable experimental effort and can lead to significant waste. Linking the structure of a chiral compound to a CSP/MP system suitable for its enantioseparation can be an effective solution to this problem. In this study, we evaluate algorithmic tools for this purpose. Our proposed consensus model, which uses multiple optimized artificial neural networks (ANNs), shows potential as an intelligent recommendation system (IRS) for ranking chromatographic systems suitable for the enantioresolution of chiral compounds with different molecular structures. To evaluate the IRS potential in a proof-of-concept stage, 56 structural descriptors for 56 structurally unrelated chiral compounds across 14 different families are considered. Chromatographic systems under study comprise 7 cellulose and amylose derivative CSPs and acetonitrile or methanol aqueous MPs (14 chromatographic systems in all). The ANNs are optimized using a fit-for-purpose version of the chaotic neural network algorithm with competitive learning (CCLNNA), a novel approach not previously applied in the chemical domain. CCLNNA is adapted to define the inner ANN complexity and perform feature selection of the structural descriptors. A customized target function evaluates the correctness of recommending the appropriate CSP/MP system. The ANN-consensus model exhibits no advisory failures and requires only an experimental attempt to verify the IRS recommendation for complete enantioresolution. This outstanding performance highlights its potential to effectively resolve this problem.

## Introduction

The study of the implications of chirality for life is an area of active research and debate due to the large number of chiral molecules that are part of living organisms and our daily lives. To conduct these studies, analytical methods for separating enantiomers of chiral molecules are paramount.

The use of chiral stationary phases (CSPs) in different chromatographic techniques is undoubtedly a good option for enantiomer separation. Among the wide variety of commercially available CSPs, those based on amylose and cellulose polysaccharide derivatives are the preferred choice for the enantioseparation of chiral compounds.^1^ These CSPs, which are widely used in both supercritical fluid chromatography (SFC) and high-performance liquid chromatography (HPLC), enable the separation of the enantiomers of a variety of compounds.^1^ SFC, with its higher efficiency, reduced environmental impact, faster separations, and ease of mobile phase removal, is a competitive choice for preparative-scale enantiomer separations. However, SFC has fewer alternative separation mechanisms compared to HPLC, and its mobile phase selection is relatively limited, offering fewer opportunities to manipulate separation selectivity based on its composition.^1^ In HPLC, separations can be carried out in the normal phase (NPLC), reversed-phase (RPLC), hydrophilic interaction liquid chromatography (HILIC), and polar organic modes. At the analytical scale, RPLC and HILIC have advantages in the analysis of aqueous samples (e.g., biological, pharmaceutical, and environmental) and in coupling with mass spectrometry detection.^2^

Numerous studies have addressed the fundamentals of enantioseparation mechanisms involving polysaccharide-based CSPs.^3−5^ Despite the advances, finding the right CSP/mobile phase (MP) combination for the separation of a pair of enantiomers remains a challenge;^6−8^ in fact, chiral separations are considered one of the most challenging of all analytical separations. The most common strategy is to test a series of CSP/MP combinations, a trial-and-error approach that often requires considerable experimental and economic effort that can lead to significant waste,^6−9^ which contradicts green chemistry principles and sustainable development goals (SDGs). Alternatively, quantitative structure-enantioselective retention relationships (QSERR) have emerged as a useful sustainable strategy to select a suitable CSP/MP combination in chiral HPLC optimization processes.^10^ For polysaccharide-based CSPs, QSERR have been constructed using chemometric tools, which allow the prediction of enantioselective parameters.^11−18^

In recent years, artificial intelligence (AI) has experienced significant growth, leading to significant advances in various fields of science and technology.^19,20^ In analytical chemistry, AI has been applied to optimize and interpret data in various analytical techniques including “omics” analysis, biosensors, or microfluidics.^21^ In chromatography, AI has facilitated the identification and quantification of compounds^21,22^ and method optimization.^23,24^

Among AI methodologies, artificial neural networks (ANNs) are a flexible machine learning approach capable of modeling complex/nonlinear relationships between input and response variables. ANNs were designed to learn from a training data set using a neural architecture that consists of interconnected artificial neurons (calculation units) arranged in several layers. However, due to the inner complexity of ANN, it is difficult to extract information on how models work to predict the output from input variables. Of notable interest is the use of ANNs to model quantitative retention-structure relationships.^25−28^ In chiral liquid chromatography, ANNs have been used to predict enantioselective parameters using different CSPs.^23,29−34^ Our research group, for the first time, used ANN to quantitatively estimate the enantioresolution (*R*_s_) of a heterogeneous set of chiral molecules.^32^

Some previous attempts to recommend the most suitable CSP for the enantioseparation of a given compound have been found in the literature.^15,33−35^ These approaches include rule-based expert system,^35^ random forest,^15^ and neural networks^33,34^ and rely on large databases (e.g., ChirBase, data taken from the literature) to predict enantioselectivity^15,34^ and retention times.^31^ In these studies, the CSP selection is based on the separation probability^33^ or on the CSPs’ enantioselectivity.^15,34^ Although these models have demonstrated some degree of success, they exhibit some limitations, mainly related to the quality of the data. Additionally, in some cases, the effects of the mobile phase are not considered,^15,34^ so predictions and recommendations are mainly limited to CSPs. Conversely, none of the suggested models were purpose-built to recommend chromatographic systems using *R*_s_ data.

In a scientific-mathematical problem, where a response depends on several variables, optimization aims to identify which are relevant (process of feature selection), or their appropriate values, to maximize or minimize an objective function related to the “goodness” of the response.^36^ Among different optimization strategies, metaheuristic optimization algorithms have shown good performance in optimizing complex and nonlinear problems.^36^ Neural network algorithm (NNA) is a new metaheuristic algorithm inspired by ANNs with high global search ability.^37^ However, this algorithm exhibits slow and premature (local) convergence when applied to complex problems. Recently, Zhang reported an improved NNA, named CCLNNA (chaotic neural network algorithm with competitive learning), to overcome these drawbacks.^38^ CCLNNA divides the population of solutions (vectors of parameters to optimize) into excellent and common subpopulations to improve the global search capability and incorporates learning strategies such as the average position of the current population and the chaotic map, to avoid premature convergence. The modifications introduced in NNA significantly improve the optimization performance, making CCLNNA a powerful algorithm for solving complex multimodal optimization problems compared to other competing algorithms.^38^

This study presents a novel AI-based approach designed to provide a cost-effective and sustainable solution as an alternative to traditional trial-and-error methods commonly used in the selection of chromatographic systems for chiral analysis. The main objective is to evaluate the effectiveness of a consensus model composed of multiple ANNs as an intelligent recommendation system (IRS) for chiral chromatography systems. The IRS provides CSP/MP recommendations for the enantioseparation of chiral compounds based on a reduced set of their structural descriptors. The specific objectives to achieve this central goal are (i) To optimize the CSP/MP recommendation correctness of independent ANNs, through an adapted CCLNNA algorithm. (ii) To collect those with the best recommending capability for appropriate chromatographic systems. (iii) To develop an ANN-consensus model that prioritizes chromatographic systems to be assayed in the laboratory. (iv) To assess the potential of the recommendation model and its effectiveness. The hypothesis is that a satisfactory model potential, at the current proof-of-concept stage, could guide cost-benefit decisions regarding incorporating sufficient additional data for a final robust model. As far as we know, the CCLNNA algorithm has not been used before for feature selection or to optimize an ANN. In addition, an IRS specifically designed and optimized to directly recommend chromatographic systems has not been reported previously.

## Experimental Section

### Chromatographic and Structural Data

To obtain structure-enantioresolution relationships via ANN, we used experimental *R*_s_ data and structural descriptors for a set of 56 chiral compounds belonging to 14 structurally unrelated families (see Table S1). The *R*_s_ values comprised a total of 14 CSP/MP systems. The CSPs used were cellulose tris(3,5-dimethylphenylcarbamate) (C1); cellulose tris(3-chloro-4-methylphenylcarbamate) (C2); cellulose tris(4-methylbenzoate) (C3); cellulose tris(4-chloro-3-methylphenylcarbamate) (C4); immobilized cellulose tris(3,5-dichlorophenylcarbamate) (C5); amylose tris(3,5-dimethylphenylcarbamate) (A1); and immobilized amylose tris(3-chloro-5-methylphenylcarbamate) (A3). All columns (3 μm, 150 × 4.6 mm i.d.) were acquired from Phenomenex (Torrance, CA). For each CSP, the MPs assayed were composed of binary hydro-organic mixtures, which included NH_4_HCO_3_ (5 mM, pH = 8.0) and either acetonitrile (abbreviated as “a”) or methanol (abbreviated as “m”). The proportions of these mixtures varied: 20–98% v/v for acetonitrile (resulting in 10 different MPs) and 30–90% v/v for methanol (resulting in 7 different MPs); see the Supporting Information. The CSP/MP systems were numbered and coded as 1 (C1a), 2 (C2a), 3 (C3a), 4 (C4a), 5 (C5a), 6 (C1m), 7 (C2m), 8 (C3m), 9 (C4m), 10 (C5m), 11 (A1a), 12 (A3a), 13 (A1m), and 14 (A3m).

Separations were performed at a flow rate of 1 mL·min^–1^ and a temperature of 25 °C. Most of the data were taken from previous papers,^18,32,39^ except those corresponding to CSPs A1 and A3 with MPs containing more than 80% acetonitrile and all methanol MPs, which were obtained experimentally in this work. All experiments for compounds *N* = 29, 34, 37, 51, and 56 with CSPs A1 and A3; and for compounds *N* = 4 and 26 with C2, C3, C4, and C5 cellulose, CSPs were also performed in this work, as well as experiments for omeprazole and tebuconazole (external test compounds). For each compound and CSP, among the different experimental *R*_s_ values obtained with the several MPs tested, the maximum *R*_s_ value was considered in this work. The experimental maximum *R*_s_ values were categorized using the binary codes as 0 (*R*_s_ < 1.5; null or incomplete enantioresolution) and 1 (*R*_s_ ≥ 1.5; complete enantioresolution) and constitute the response matrix (**T**; see Table S1).

The descriptor matrix (**X**) consisted of 56 structural descriptors (see Table S2). These descriptors include 7 chiral carbon-related parameters (**x**_**1**_–**x**_**7**_) obtained as the number of atoms/groups bonded to the chiral atom (C*); e.g., C*a (**x**_**4**_) corresponds to the number of aliphatic groups directly bonded to the chiral atom. The rest (molecular descriptors and topological and hydrophobicity parameters) were estimated descriptors from MarvinSketch (©ChemAxon Ltd., version 21.8.0) and ChemSketch (©Advanced Chemistry Development, Inc., version 2020.2.0) software. Examples of these parameters are the molar mass (**x**_**8**_), the number of aromatic groups with 1,4 substitutions (**x**_**49**_), the number of amino tertiary groups in aliphatic cycles (**x**_**53**_), among others. **X**-variables were autoscaled as a pretreatment in this work.

### ANN Nomenclature and Strategies

In this study, ANN architecture design focused on regression. The input layer consisted of a data matrix of known structural descriptors (**X**) for the set of compounds (Table S2), while matrices of known **T** values (Table S1; training stage) or predicted *R*_s_ response values (**Yc**; prediction stage) were positioned in the output layer. We tested ANNs with a maximum of 2 hidden layers, with k neurons in the first hidden layer, between 1 and 30, and kk neurons in the second hidden layer, between 0 and 30 (0 means only one hidden layer). As in any regression model, ANN predictions (**Yc**) initially appeared as continuous data but were later converted into discrete values (**Y**; 0 or 1) according to the criteria depicted in Table S3. Comparing the **Y** values with **T** allowed us to determine whether the ANN recommendation was correct or not.

To preserve the ANN representativeness, given the limited number of available compounds, we decided to use just 6 compounds for validation (to mitigate ANN overfitting) and 2 test compounds for prediction. The remaining 48 compounds were reserved for training the ANN. The adapted CCLNNA algorithm automatically selected the validation and test compounds. In addition, as part of the optimization of the ANN, CCLNNA also determined the internal network complexity (k, kk) and included a feature selection process to reduce the number of descriptors (ND) for each ANN.

### Software and Calculations

MATLAB R2022b (Mathworks) was used for adapting/programming the algorithms, in conjunction with its Deep Learning Toolbox library that contains algorithms for creating and training ANNs, as well as the original CCLNNA version.^38^ CCLNNA operates on solution vectors, initially defined as continuous parameters to be optimized. In this case, our adapted CCLNNA version includes a necessary modification of the original CCLNNA algorithm to convert the continuous values of each solution vector into integer values. As solution vector, the following 66 indexes were used: 8 indexes corresponding to the selected validation and test compounds (*N* values in Table S1), 2 indexes corresponding to the inner ANN architecture (k and kk between the above indicated limits), and 56 indexes corresponding to the descriptors, whose values in the 0–1 range were converted to 0 or 1 (threshold value = 0.8) indicating the presence or absence, respectively, of any descriptor into the model.

An additional modification to the CCLNNA algorithm includes maximizing a target function, named PScoreo (penalized score considering overfitting), to achieve the objectives of this study. The PScoreo for the ANN model is a mean value calculated from the individual contributions of each compound *i* (PScoreo_*i*_) as follows:
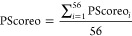
1

The following equation is proposed for calculating PScoreo_*i*_
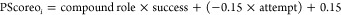
2where “Compound role” has a value equal to 1, 2, or 3 depending on whether compound *i* is assigned to the training, validation, and test subset, respectively. The inclusion of the term “Compound role” in PScoreo_*i*_ aimed to mitigate overfitting by penalizing errors concentrated on test and validation compounds.

“Success” and “Attempt” values were computed based on the criteria outlined in Table S3. For a given compound *i*, the optimized ANN provided a **Yc** output associated with a given CSP/MP system. We assigned a coded **Y** value of 1 (if **Yc** ≥ 0.5) or 0 (if **Yc** ≤ 0.25). For intermediate **Yc** values (0.25 < **Yc** < 0.5), both **Y** values (0 or 1) were considered. A “Success” value was assigned based on the agreement between the **Y** and **T** values. “Attempt” refers to the number of attempts required to achieve a Success >0. For each compound *i*, up to three CSP/MP system recommendations (R_1_, R_2_, and R_3_) were considered. These corresponded to the three highest **Yc** values predicted by the ANN in descending order.

Note that the assignment of “Success” and “Attempt” values is based on a personal assessment, similar to how an analyst in the laboratory would evaluate the usefulness of a given recommendation. The rules in Table S3 were used solely for optimizing each ANN. Other customized equations and rules could be considered, but such exploration is beyond the scope of this proof-of-concept stage.

Two main CCLNNA parameters were configured: the maximum number of iterations (MaxIt) varied from 150 to 300, and the solution population size (nPop) ranged from 50 to 150.

## Results and Discussion

### Intelligent Recommendation System (IRS)

An IRS specifically designed to assist in CSP/MP system selection can replace traditional trial-and-error methods, human recommenders (if they exist), and models focused only on predictive or classifying abilities. The IRS would learn from molecular descriptors and enantioresolution data to provide hierarchical CSP/MP recommendations (R_1_ > R_2_ > R_3_) or to signal unfeasible enantioresolution. The IRS’s strength lies in optimizing its recommendation capability. This concept could spur further research for a broader CSP/MP-IRS. Neural network arrangements (CCLNNA, ANNs) seem to be suitable for the expected complexity. See the Table S4 for further exploration of the topic.

### ANNs Optimization

The optimization of ANNs (to maximize the PScoreo target function) was conducted using the CCLNNA algorithm, as outlined in the Experimental Section. A total of 30 CCLNNA-ANN processes were executed. We arranged the outcomes of the generated ANNs by descending the PScoreo values. For instance, Table 1 presents the top 11 ANNs ranked by their PScoreo.

**Table 1:** Main Features of the Top 11 ANNs with the Highest PScoreo Values^a^

PScoreo	extra attempts	validation objects						test objects		k	kk	ND	ANN-consensus model^b^
117.7	2	4	16	**23**	31	38	56	14	**46**	14	28	**13**	**ANN1**
117.6	3	4	20	**23**	33	**41**	**51**	13	43	28	**0**	**16**	**ANN2**
117.5	3	**5**	**15**	**23**	**29**	**41**	**51**	10	43	30	**0**	20	
117.3	3	**5**	**15**	**23**	34	42	55	14	**46**	23	29	23	
117.2	3	**5**	17	25	**29**	36	52	11	**46**	23	18	**8**	**ANN3**
116.6	5	1	**15**	**23**	**29**	38	56	8	43	30	**0**	**16**	
116.6	5	1	**15**	26	35	36	55	8	**46**	30	**0**	18	
116.1	4	3	**15**	27	31	37	50	9	49	18	**0**	**13**	**ANN4**
115.9	4	1	**15**	**23**	**29**	**41**	**51**	9	43	30	**0**	28	
115.9	5	1	16	**23**	**29**	**41**	53	13	43	29	30	18	
115.8	4	1	16	**23**	31	38	50	11	47	22	24	**4**	**ANN5**

a See further details in the Experimental Section.

b ANNs selected to evaluate a consensus model as IRS.

Table S1 shows that 44 of the compounds studied are completely enantioresolved using one or more CSP/MP systems, while 12 compounds cannot be enantioresolved with any of the systems tested. This implies that an ideal IRS would perform 44 “attempts”, which means that the first recommendation (R_1_) would be sufficient to achieve complete enantioresolution on the first “attempt”, as nonenantioresolved compounds do not require an “attempt” (see Table S3). Thus, for each ANN, it is possible to calculate the difference between the total attempts required to achieve complete enantioresolution and this minimum value (44). Table 1 also shows the number of “extra attempts” for the top 11 ANNs.

The main discovery from the examination of the 30 ANNs was the small number of failures in ANN recommendations (only 5 ANNs failed to provide a correct recommendation for one or two compounds within the 3 allowed attempts). This suggests the high effectiveness of the CCLNNA-ANN combination. It was noted that ANNs with a PScoreo value >112 consistently provide correct recommendations for all compounds. On the other hand, the “extra attempt” values exhibited significant variability, ranging from 2 to 20 for the 30 ANNs. Additional insights were as follows: (i) An inverse correlation between the PScoreo and the “extra attempts” values, as it could be expected. Such a relationship became more evident as PScoreo raised. (ii) PScoreo did not show clear relationships with nPop, ANN internal complexity (k + kk), or ND. (iii) A modest positive correlation between PScoreo and MaxIt, although some satisfactory ANNs were achieved with only 150 iterations.

Compared with the 30 ANNs, the 11 ANNs in Table 1 exhibited PScoreo > 115, suggesting a low probability of failure for new compounds, and the lowest “extra attempt” values (≤5), suggesting low experimental effort in a future application. However, they present differences in their complexity. The simplest ANNs with one hidden layer (kk = 0; in bold in Table 1) and a small number of descriptors (ND ≤ 16 in bold in Table 1) are expected to be more robust. On the other hand, the CCLNNA automatically selected different validation and test object subsets for each ANN (the most frequent are also bolded in Table 1).

### ANNs Consensus Model as IRS

Despite the impressive performance and reasonable number of “extra attempts” by the top-performing individual ANNs (referenced in Table 1), we opted for an ANN-consensus model to establish a more reliable IRS. This model combines the recommendations made by multiple ANNs to provide the predominant one as a single recommendation. The rationale behind this approach is to reinforce the reliability of the recommendations while trying to minimize the number of “extra attempts”. From the available options, we chose to evaluate an ANN-consensus model comprising the five ANNs from Table 1 with the lowest number of descriptors (ND ≤ 16) and “extra attempts” ≤ 4. These five ANNs are highlighted in Table 1 (ANN1–ANN5). The joint decisions of these five ANNs would consensually determine the final CSP/MP system recommendation (IRS recommendation). The simplest option was to look at the first recommendation (R_1_) from each ANN and select the most frequent of the five R_1_ as the IRS recommendation.

Table 2 shows the CSP/MP systems corresponding to R_1_ provided by each of the five ANNs comprising the ANN-consensus model for those compounds having a most frequent R_1_ value (i.e., IRS recommendation). Table 2 also shows the estimated (i.e., **Y** estimation) and experimental (i.e., **T**; Table S1) categorical enantioresolution values corresponding to IRS recommendations. CSP/MP system = 0 is used for those compounds with **Y** = 0 values.

**Table 2:** CSP/MP Systems Corresponding to the R_1_ Recommendation for the Five Selected ANNs and the IRS Recommendation (the Most Frequent R_1_) Together with Their Corresponding Categorical (**Y**) and Experimental (**T**) Enantioresolution^a^

		CSP/MP system						categorical enantioresolution	
*N*^b^	compound	ANN1	ANN2	ANN3	ANN4	ANN5	IRS	IRS (**Y**)	experimental (**T**)
1	disopyramide	12	12	12	11	11	12	1	1
2	mexiletine	0	0	0	0	0	0	0	0
3	propafenone	11	13	13	13	11	11	1	1
4	warfarin	12	5	2	11	11	11	1	1
5	nomifensine	12	4	12	8	12	12	1	1
6	citalopram	2	2	2	2	2	2	1	1
7	fluoxetine	0	0	0	13	0	0	0	0
9	trimipramine	3	3	3	8	2	3	1	1
10	bupropion	11	13	11	13	11	11	1	1
11	mianserin	8	3	3	2	3	3	1	1
13	imazalil	2	4	2	7	2	2	1	1
14	penconazole	5	5	5	4	2	5	1	1
17	metalaxyl	4	2	4	4	4	4	1	1
18	doxylamine	2	2	2	2	13	2	1	1
19	brompheniramine	0	0	0	0	0	0	0	0
20	chlorpheniramine	0	0	0	0	0	0	0	0
21	orphenadrine	1	1	6	6	6	6	1	1
22	carbinoxamine	11	13	13	11	13	13	1	1
24	terfenadine	5	5	5	5	5	5	1	1
25	cetirizine	3	3	13	3	11	3	1	1
26	fexofenadine	5	0	0	5	0	0	0	0
27	mepivacaine	2	2	5	5	2	2	1	1
28	propanocaine	2	4	4	2	2	2	1	1
29	prilocaine	13	2	13	13	13	13	1	1
30	bupivacaine	8	8	8	8	8	8	1	1
32	bicalutamide	11	12	5	14	12	12	1	1
33	pindolol	13	13	10	13	13	13	1	1
34	propranolol	13	13	13	13	13	13	1	1
35	metoprolol	0	0	0	0	0	0	0	0
36	acebutolol	13	13	11	13	13	13	1	1
37	atenolol	0	0	0	0	0	0	0	0
38	salbutamol	0	0	0	0	0	0	0	0
39	timolol	0	0	0	0	0	0	0	0
40	bambuterol	0	0	0	0	0	0	0	0
41	isoprenaline	12	12	12	12	12	12	1	1
42	orciprenaline	12	3	12	12	12	12	1	1
43	clenbuterol	2	4	4	4	2	4	1	1
44	terbutaline	2	5	5	5	5	5	1	1
45	verapamil	0	14	0	0	0	0	0	0
46	felodipine	2	2	2	8	8	2	1	1
47	cinildipine	10	10	10	10	10	10	1	1
48	procyclidine	0	0	0	14	2	0	0	0
49	trimeprazine	8	8	3	8	8	8	1	1
50	etopropazine	3	3	3	3	8	3	1	1
52	thioridazine	3	3	3	3	3	3	1	1
54	methadone	3	3	8	3	3	3	1	1
56	lansoprazole	4	5	11	4	2	4	1	1

a See the Experimental Section to identify the CSP/MP systems.

b Compounds not listed have an undefined consensus outcome.

The comparison of **Y** and **T** data allows us to check the performance of the ANN-consensus model. For instance, for compound *N* = 1, three ANNs recommend the CSP/MP system 12 (Amylose3/acetonitrile, A3a), while the remaining two suggest system 11 (Amylose1/acetonitrile, A1a). The IRS recommendation would be system 12 (i.e., the IRS predicts complete enantioresolution with this chromatographic system, **Y** = 1), consistent with the experimental observation in Table S1. Note that system 11 would also produce the correct output. In a future application of the model for selecting the appropriate chromatographic system, the analyst would need only one confirmatory laboratory test (the minimum necessary) to verify the IRS recommendation. In this case, “extra attempts” would be 0.

Compound *N* = 2, for which the model suggests no enantioresolution in any of the CSP/MP systems (R_1_ = 0 for all of the ANNs), represents the opposite case. The prediction (**Y** = 0) agrees with the experimental observation (**T** = 0 in all CSP/MP systems, see Table S1). In this case, no confirmatory tests are necessary (and would not be carried out in practice); thus, “extra attempts” would be 0.

Given that the IRS recommendation for all compounds in Table 2 is correct (**Y** = **T**; in the first attempt), the number of “extra attempts” required is zero, demonstrating the outstanding performance of the ANN-consensus model.

However, Table 2 includes 84% of the compounds. The rest of the compounds (*N* = 8, 12, 15, 16, 23, 31, 51, 53, 55) present decision-making ambiguities due to the lack of a dominant R_1_ recommendation (see Table 3). This can be particularly problematic for cases such as compounds *N* = 12, 16, and 31, where the five ANN R_1_ recommendations differ. To address these challenges, we devised a different approach for the ANN-consensus model, incorporating the second recommendation (R_2_) from each ANN. Table 3 shows the results of the R_1_ and R_2_ recommendations for compounds not included in Table 2. Additionally, it includes a new parameter determining the reliability of the IRS recommendation (on a scale from 0 to 1), using the following equation that weights R_1_ and R_2_ recommendations
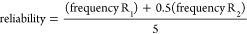
3

**Table 3:** CSP/MP Systems Corresponding to the R_1_ and R_2_ Recommendations for the Five Selected ANNs and the IRS Recommendation Together with Their Corresponding Reliability, Categorical (**Y**) and Experimental (**T**) Enantioresolution^a^

		CSP/MP system												categorical enantioresolution	
		ANN1		ANN2		ANN3		ANN4		ANN5					
*N*	compound	R_1_	R_2_	R_1_	R_2_	R_1_	R_2_	R_1_	R_2_	R_1_	R_2_	IRS	reliability	IRS (**Y**)	experimental (**T**)
8	viloxazine	2	13	11	9	11	2	7	2	7	2	2	0.5	1	1
12	benalaxyl	11	12	5	11	4	12	12	5	14	11	11, 12	0.4	1	1
15	hexaconazole	2	4	7	11	2	13	7	9	13	2	2	0.5	1	1
16	myclobutanil	1	8	12	6	3	13	4	7	13	2	13	0.3	1	1
23	hydroxyzine	11	3	11	1	13	11	3	11	3	11	11	0.7	1	1
31	aminoglutethimide	2	1	9	11	3	5	7	9	12	7	7	0.3	1	1
51	promethazine	8	3	3	10	12	3	3	11	8	3	3	0.7	1	1
53	pantoprazole	5	2	5	14	4	9	12	4	4	12	4	0.5	1	1
55	rabeprazole	5	12	5	12	12	4	14	12	12	2	12	0.7	1	1

a See the Experimental Section to identify the CSP/MP systems.

The most reliable recommendation in Table 3 was selected as the IRS recommendation. Again, we found excellent results in terms of IRS recommendation correctness (there is an agreement between **Y** and **T** values in all cases) and experimental effort minimization (zero “extra attempts”). For compound *N* = 12, two IRS recommendations (CSP/MP = 11, 12) with the same reliability were obtained; anyway, both are correct and would imply zero “extra attempts”.

Such approaches and equations can significantly aid the decision-making process and may be customized if convenient by the analyst. For instance, the first three recommendations could be incorporated (e.g., in cases such as compound *N* = 12) to try to increase the reliability and to eliminate the human factor from the decision-making process.

An additional benefit of the ANN-consensus model is that it can assess which structural descriptors are most frequently involved in the assessment, making the consensus model a sort of indirect method for establishing the relative importance of descriptors (see the Supporting Information; Figure S1).

### Application Examples of the IRS in a Proof-of-Concept Context

**Figure 1:**
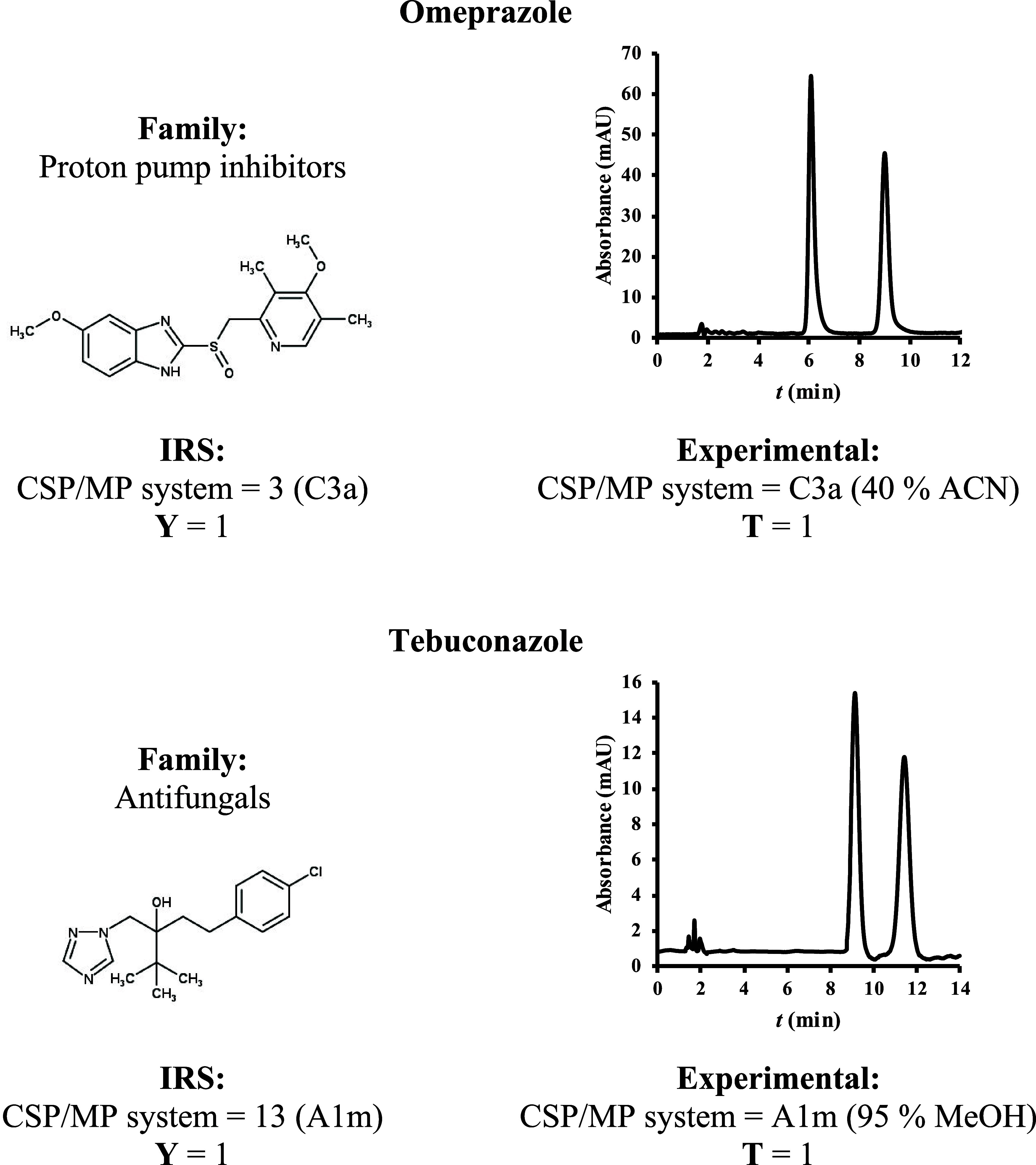
Application examples of the IRS for the external test compounds omeprazole and tebuconazole and experimental results.

The current IRS was utilized on two external test compounds (omeprazole and tebuconazole; unknown **T**), belonging to families studied, just with the goal of further reinforcing its potential. The recommended IRS CSP/MP systems for these compounds were later experimentally confirmed (i.e., **Y** = **T**; Figure 1), underscoring its potential and advocating for continued research to move beyond the proof-of-concept stage.

## Conclusions

We propose new algorithmic tools capable of recommending chiral chromatography systems for the enantioresolution of a heterogeneous set of compounds from selected structural molecular descriptors. For the first time (to the best of our knowledge), a CCLNNA optimization (adapted) algorithm has been combined with ANN; thus, two different neural network approaches are merged (CCLNNA-ANN). The approach developed includes autonomous CCLNNA strategy, guided by the own ANN outputs, involving: (i) compounds subsets selection, (ii) ANN architecture optimization, and (iii) feature selection on descriptors. The overall strategy has been designed for comparing different ANNs (according to a new fit-for-purpose customizable PScoreo equation), based on the efficiency on correctness (recommended CSP/MP system) and minimal experimental verification effort.

The following conclusions can be drawn from the results obtained in this work, limited to its aim: (i) The optimization of fit-for-purpose ANNs (directed to rank the chromatographic systems for complete enantioresolution of a given neutral or basic chiral compound based on its structure) by means of the adapted CCLNNA algorithm has the potential to be a practical and effective bet. (ii) The CCLNNA-ANN approach provides excellent results with a very low number of assessment failures. (iii) A limited small number of experimental tests beyond the planned ones (extra attempts) to verify the recommendations allows filtering out the most suitable ANNs. (iv) Best ANNs can form a consensus model, contributing to increase the potential of the proposed strategy. The first recommendation (R_1_) of each ANN forming the IRS recommendation provides the right CSP/MP system for most of the compounds studied. In the case of ambiguity, the second recommendation (R_2_) of each ANN facilitates the decision-making process. For the case under study, the ANN-consensus model has an outstanding performance since there is a full agreement between the IRS recommendation and the experimental results. (v) The IRS (combining CCLNNA-ANN and ANN-consensus model strategies), in the framework of artificial intelligence, has proven enough potential to provide a simple solution to a highly complex problem (a tool to be used by analysts interested in the enantioseparation a given chiral compound). Thus, the collection of more experimental data (more compounds from more families, more chiral stationary phases, and mobile phases, maybe more descriptors) is encouraged to derive a single ANN or an ANN-consensus model able to recommend the suitable chiral chromatographic system to any future compound, accompanied by an improved probability of correctness.

## Supporting Information Available

Compounds, family, and binary codes for categorical enantioresolution (matrix **T**) for the 14 CSP/MP systems studied; composition of the mobile phases; structural descriptors used for modeling; customized criteria applied during CCLNNA optimization; exploration of the topic intelligent recommendation system (IRS); and an approximation to the relative importance of the molecular descriptors. (PDF)

## References

[ref1] Chankvetadze B. Polysaccharide-Based Chiral Stationary Phases for Enantioseparations by High-Performance Liquid Chromatography: An Overview. In Chiral Separations. Methods in Molecular Biology; Scriba G. K. E., Ed.; Humana Press: New York, NY, 2019. 10.1007/978-1-4939-9438-0_6 31069731

[ref2] Tarafder A.; Miller L. Chiral chromatography method screening strategies: Past, present and future. J. Chromatogr. A 2021, 1638, 461878 10.1016/j.chroma.2021.461878. 33477025

[ref3] Scriba G. K. E. Chiral recognition in separation sciences. Part I: Polysaccharide and cyclodextrin selectors. TrAC, Trends Anal. Chem. 2019, 120, 115639 10.1016/j.trac.2019.115639.

[ref4] Kasat R. B.; Wee S. Y.; Loh J. X.; Franses E. I.; Wang N.-H. L. Effect of the solute molecular structure on its enantioresolution on cellulose tris(3,5-dimethylphenylcarbamate). J. Chromatogr. B 2008, 875, 81–92. 10.1016/j.jchromb.2008.06.045. 18635409

[ref5] Varfaj I.; Di Michele A.; Ianni F.; Saletti M.; Anzini M.; Barola C.; Chankvetadze B.; Sardella R.; Carotti A. Enantioseparation of novel anti-inflammatory chiral sulfoxides with two cellulose dichlorophenylcarbamate-based chiral stationary phases and polar-organic mobile phase(s). J. Chromatogr. Open 2021, 1, 100022 10.1016/j.jcoa.2021.100022.

[ref6] Losacco G. L.; Wang H.; Haidar Ahmad I. A.; DaSilva J.; Makarov A. A.; Mangion I.; Gasparrini F.; Lämmerhofer M.; Armstrong D. W.; Regalado E. L. Enantioselective UHPLC Screening combined with in silico modeling for streamlined development of ultrafast enantiopurity assays. Anal. Chem. 2022, 94, 1804–1812. 10.1021/acs.analchem.1c04585. 34931812

[ref7] Lin J.; Tsang C.; Lieu R.; Zhang K. Method screening strategies of stereoisomers of compounds with multiple chiral centers and a single chiral center. J. Chromatogr. A 2020, 1624, 461244 10.1016/j.chroma.2020.461244. 32540081

[ref8] Barhate C. L.; Joyce L. A.; Makarov A. A.; Zawatzky K.; Bernardoni F.; Schafer W. A.; Armstrong D. W.; Welch C. J.; Regalado E. L. Ultrafast chiral separations for high throughput enantiopurity analysis. Chem. Commun. 2017, 53, 509–512. 10.1039/C6CC08512A. 27872920

[ref9] Welch C. J. Microscale chiral HPLC in support of pharmaceutical process research. Chirality 2009, 21, 114–118. 10.1002/chir.20625. 18655169

[ref10] De Gauquier P.; Vanommeslaeghe K.; Vander Heyden Y.; Mangelings D. Modelling approaches for chiral chromatography on polysaccharide-based and macrocyclic antibiotic chiral selectors: A review. Anal. Chim. Acta 2022, 1198, 338861 10.1016/j.aca.2021.338861. 35190117

[ref11] Khater S.; Lozac’h M. A.; Adam I.; Francotte E.; West C. Comparison of liquid and supercritical fluid chromatography mobile phases for enantioselective separations on polysaccharide stationary phases. J. Chromatogr. A 2016, 1467, 463–472. 10.1016/j.chroma.2016.06.060. 27378250

[ref12] Barfeii H.; Garkani-Nejad Z. A comparative QSRR study on enantioseparation of ethanol ester enantiomers in HPLC using multivariate image analysis, quantum mechanical and structural descriptors. J. Chin. Chem. Soc. 2017, 64, 176–187. 10.1002/jccs.201600253.

[ref13] Pisani L.; Rullo M.; Catto M.; de Candia M.; Carrieri A.; Cellamare S.; Altomare C. D. Structure–property relationship study of the HPLC enantioselective retention of neuroprotective 7-[(1-alkylpiperidin-3-yl) methoxy] coumarin derivatives on an amylose-based chiral stationary phase. J. Sep. Sci. 2018, 41, 1376–1384. 10.1002/jssc.201701442. 29419937

[ref14] Luo C.; Hu G.; Huang M.; Zou J.; Jiang Y. Prediction on separation factor of chiral arylhydantoin compounds and recognition mechanism between chiral stationary phase and the enantiomers. J. Mol. Graphics Model. 2020, 94, 107479 10.1016/j.jmgm.2019.107479. 31671366

[ref15] Sheridan R.; Schafer W.; Piras P.; Zawatzky K.; Sherer E. C.; Roussel C.; Welch C. J. Toward structure-based predictive tools for the selection of chiral stationary phases for the chromatographic separation of enantiomers. J. Chromatogr. A 2016, 1467, 206–213. 10.1016/j.chroma.2016.05.066. 27318509

[ref16] Piras P.; Sheridan R.; Sherer E. C.; Schafer W.; Welch C. J.; Roussel C. Modeling and predicting chiral stationary phase enantioselectivity: an efficient random forest classifier using an optimally balanced training dataset and an aggregation strategy. J. Sep. Sci. 2018, 41, 1365–1375. 10.1002/jssc.201701334. 29383846

[ref17] Martín-Biosca Y.; Escuder-Gilabert L.; Medina-Hernández M. J.; Sagrado S. Modelling the enantioresolution capability of cellulose tris (3, 5-dichlorophenylcarbamate) stationary phase in reversed phase conditions for neutral and basic chiral compounds. J. Chromatogr. A 2018, 1567, 111–118. 10.1016/j.chroma.2018.06.061. 29960736

[ref18] Pérez-Baeza M.; Escuder-Gilabert L.; Martín-Biosca Y.; Sagrado S.; Medina-Hernández M. J. Comparative modelling study on enantioresolution of structurally unrelated compounds with amylose-based chiral stationary phases in reversed phase liquid chromatography-mass spectrometry conditions. J. Chromatogr. A 2020, 1625, 461281 10.1016/j.chroma.2020.461281. 32709332

[ref19] Ayres L. B.; Gomez F. J. V.; Linton J. R.; Silva M. F.; Garcia C. D. Taking the leap between analytical chemistry and artificial intelligence: A tutorial review. Anal. Chim. Acta 2021, 1161, 338403 10.1016/j.aca.2021.338403. 33896558

[ref20] Baum Z. J.; Yu X.; Ayala P. Y.; Zhao Y.; Watkins S. P.; Zhou Q. Artificial intelligence in chemistry: current trends and future directions. J. Chem. Inf. Model. 2021, 61, 3197–3212. 10.1021/acs.jcim.1c00619. 34264069

[ref21] Rial R. C. AI in analytical chemistry: Advancements, challenges, and future directions. Talanta 2024, 274, 125949 10.1016/j.talanta.2024.125949. 38569367

[ref22] Singh Y. R.; Shah D. B.; Kulkarni M.; Patel S. R.; Maheshwari D. G.; Shah J. S.; Shah S. Current trends in chromatographic prediction using artificial intelligence and machine learning. Anal. Methods 2023, 15, 2785–2797. 10.1039/D3AY00362K. 37264667

[ref23] Korany M. A.; Mahgoub H.; Fahmy O. T.; Maher H. M. Application of artificial neural networks for response surface modelling in HPLC method development. J. Adv. Res. 2012, 3, 53–63. 10.1016/j.jare.2011.04.001.

[ref24] Abba S. I.; Usman A. G.; Selin I. Simulation for response surface in the HPLC optimization method development using artificial intelligence models: A data-driven approach. Chemom. Intell. Lab. Syst. 2020, 201, 104007 10.1016/j.chemolab.2020.104007.

[ref25] D’Archivio A. A.; Giannitto A.; Maggi M. A.; Ruggieri F. Cross-column retention prediction in reversed-phase high performance liquid chromatography by artificial neural network modelling. Anal. Chim. Acta 2012, 717, 52–60. 10.1016/j.aca.2011.12.047. 22304815

[ref26] Fatemi M. H.; Abraham M. H.; Poole C. F. Combination of artificial neural network technique and linear free energy relationship parameters in the prediction of gradient retention times in liquid chromatography. J. Chromatogr. A 2008, 1190, 241–252. 10.1016/j.chroma.2008.03.021. 18395736

[ref27] Barron L. P.; McEneff G. L. Gradient liquid chromatographic retention time prediction for suspect screening applications: A critical assessment of a generalised artificial neural network-based approach across 10 multi-residue reversed-phase analytical methods. Talanta 2016, 147, 261–270. 10.1016/j.talanta.2015.09.065. 26592605

[ref28] Ukić Š.; Novak M.; Vlahović A.; Avdalović N.; Liu Y.; Buszewski B.; Bolanča T. Development of gradient retention model in ion chromatography. Part II: artificial intelligence QSRR approach. Chromatographia 2014, 77, 997–1007. 10.1007/s10337-014-2654-4.

[ref29] Boronová K.; Lehotay J.; Hroboňová K.; Armstrong D. W. Study of physicochemical interaction of aryloxyaminopropanol derivatives with teicoplanin and vancomycin phases in view of quantitative structure-property relationship studies. J. Chromatogr. A 2013, 1301, 38–47. 10.1016/j.chroma.2013.05.046. 23809846

[ref30] Szaleniec M.; Dudzik A.; Pawul M.; Kozik B. Quantitative structure enantioselective retention relationship for high-performance liquid chromatography chiral separation of 1-phenylethanol derivatives. J. Chromatogr. A 2009, 1216, 6224–6235. 10.1016/j.chroma.2009.07.002. 19631329

[ref31] Suzuki T.; Timofei S.; Iuoras B. E.; Uray G.; Verdino P.; Fabian W. M. F. Quantitative structure-enantioselective retention relationships for chromatographic separation of arylalkylcarbinols on Pirkle type chiral stationary phases. J. Chromatogr. A 2001, 922, 13–23. 10.1016/S0021-9673(01)00921-9. 11486858

[ref32] Pérez-Baeza M.; Martín-Biosca Y.; Escuder-Gilabert L.; Medina-Hernández M. J.; Sagrado S. Artificial neural networks to model the enantioresolution of structurally unrelated neutral and basic compounds with cellulose tris(3,5-dimethylphenylcarbamate) chiral stationary phase and aqueous-acetonitrile mobile phases. J. Chromatogr. A 2022, 1672, 463048 10.1016/j.chroma.2022.463048. 35436687

[ref33] Xu H.; Lin J.; Zhang D.; Mo F. Retention time prediction for chromatographic enantioseparation by quantile geometry-enhanced graph neural network. Nat. Commun. 2023, 14, 3095 10.1038/s41467-023-38853-3. 37248214 PMC10227049

[ref34] Hong Y.; Welch C. J.; Piras P.; Tang H. Enhanced structure-based prediction of chiral stationary phases for chromatographic enantioseparation from 3D molecular conformations. Anal. Chem. 2024, 96, 2351–2359. 10.1021/acs.analchem.3c04028. 38308813

[ref35] Bryant C.; Adam A.; Taylor D.; Rowe R. Towards an expert system for enantioseparations: induction of rules using machine learning. Chemom. Intell. Lab. Syst. 1996, 34, 21–40. 10.1016/0169-7439(96)00016-0.

[ref36] Boyd S. P.; Vandenberghe L. Convex Optimization; Cambridge University Press: Cambridge, 2004.

[ref37] Sadollah A.; Sayyaadi H.; Yadav A. A Dynamic Metaheuristic Optimization Model Inspired by Biological Nervous Systems: Neural Network Algorithm. Appl. Soft Comput. 2018, 71, 747–782. 10.1016/j.asoc.2018.07.039.

[ref38] Zhang Y. Chaotic Neural Network Algorithm with Competitive Learning for Global Optimization. Knowledge-Based Syst. 2021, 231, 107405 10.1016/j.knosys.2021.107405.

[ref39] Pérez-Baeza M.; Escuder-Gilabert L.; Martín-Biosca Y.; Sagrado S.; Medina-Hernández M. J. Comparative study on retention behaviour and enantioresolution of basic and neutral structurally unrelated compounds with cellulose-based chiral stationary phases in reversed phase liquid chromatography-mass spectrometry conditions. J. Chromatogr. A 2022, 1673, 463073 10.1016/j.chroma.2022.463073. 35500389

