# Supplementary material for: Intelligent Recommendation Systems Powered by Consensus Neural Networks: The Ultimate Solution for Finding Suitable Chiral Chromatographic Systems?
Source: Anal Chem. 2024 Jul 10;96(29):12205–12. doi: 10.1021/acs.analchem.4c02656 (PMC11270524; doi:10.1021/acs.analchem.4c02656)
Supplement: Supplementary file 1 — ac4c02656_si_001.pdf [file ac4c02656_si_001.pdf]

SUPPORTING INFORMATION

Intelligent recommendation systems powered by consensus neural networks: The ultimate solution for finding suitable chiral chromatographic systems?

Salvador Sagrado[a,b*], Carlos Pardo-Cortina[a], Laura Escuder-Gilabert[a], María José Medina-Hernández [a], Yolanda Martín-Biosca[a*].

[a] *Departamento de Química Analítica, Universitat de València E- 46100, Burjassot, Valencia, Spain*

[b] *Instituto Interuniversitario de Investigación de Reconocimiento Molecular y Desarrollo Tecnológico (IDM), Universitat Politècnica de València, Universitat de València, Valencia, Spain*

* Corresponding author: S. Sagrado (sagrado@uv.es) Phone: 34-963544878

* Corresponding author: Y. Martín-Biosca (yolanda.martin@uv.es). Phone: 34-963543186

TABLE OF CONTENTS	Page
Table S1. Compounds, family, and binary codes for categorical enantioresolution (matrix **T**) for the 14 CSP/MP systems studied.	S-2
Compositions of the mobile phases	S-3
Table S2. Structural descriptors used for modelling.	S-4
Table S3. Customized criteria applied during CCLNNA optimization for evaluating “Success” and “Attempt”.	S-6
Table S4. Exploration of the topic Intelligent recommendation system (IRS).	S-7
Approximation to the relative importance of the molecular descriptors (Figure S1).	S-8

Table S1. Compounds, family, and binary codes for categorical enantioresolution (matrix T) for the 14 CSP/MP systems studied. T = 0 (null or partial enantioresolution; Rs < 1.5), T = 1 (complete enantioresolution; Rs ≥ 1.5). T data order corresponds to the following chromatographic systems: 1 (C1a), 2 (C2a), 3 (C3a), 4 (C4a), 5 (C5a), 6 (C1m), 7 (C2m), 8 (C3m), 9 (C4m), 10 (C5m), 11 (A1a), 12 (A3a), 13 (A1m), and 14 (A3m)

N	Compound	Family	Categorical enantioresolution (Response matrix **T**)
1	Disopyramide	Antiarrhythmics	0 0 0 0 0 0 0 0 0 0 1 1 0 1
2	Mexiletine	Antiarrhythmics	0 0 0 0 0 0 0 0 0 0 0 0 0 0
3	Propafenone	Antiarrhythmics	0 0 0 0 0 0 0 0 0 0 1 0 1 0
4	Warfarin	Anticoagulants	0 1 1 1 0 0 0 0 0 0 1 1 0 0
5	Nomifensine	Antidepressants	1 1 1 1 1 0 1 1 1 1 1 1 1 1
6	Citalopram	Antidepressants	0 1 0 0 0 0 0 0 0 0 0 0 0 0
7	Fluoxetine	Antidepressants	0 0 0 0 0 0 0 0 0 0 0 0 0 0
8	Viloxazine	Antidepressants	0 1 0 0 0 0 1 0 1 0 1 1 1 0
9	Trimipramine	Antidepressants	0 1 1 0 0 0 0 0 0 0 0 0 0 0
10	Bupropion	Antidepressants	0 0 0 0 0 0 0 1 0 0 1 1 1 0
11	Mianserin	Antidepressants	1 1 1 1 0 0 0 1 0 0 1 1 1 1
12	Benalaxyl	Antifungals	0 0 0 1 1 0 0 0 0 1 1 1 1 1
13	Imazalil	Antifungals	0 1 1 1 1 0 1 1 1 1 0 0 0 0
14	Penconazole	Antifungals	0 1 0 1 1 1 1 0 0 1 0 1 0 0
15	Hexaconazole	Antifungals	1 1 1 1 1 0 1 0 1 0 1 0 1 0
16	Myclobutanil	Antifungals	1 0 1 1 0 1 1 0 1 0 0 1 1 1
17	Metalaxyl	Antifungals	1 1 1 1 1 1 1 0 0 0 1 1 0 0
18	Doxylamine	Antihistamines	0 1 0 0 0 0 0 0 0 0 0 0 0 0
19	Brompheniramine	Antihistamines	0 0 0 0 0 0 0 0 0 0 0 0 0 0
20	Chlorpheniramine	Antihistamines	0 0 0 0 0 0 0 0 0 0 0 0 0 0
21	Orphenadrine	Antihistamines	1 0 0 0 0 1 0 0 0 0 0 0 0 0
22	Carbinoxamine	Antihistamines	0 0 0 0 0 0 0 0 0 0 1 0 1 0
23	Hydroxyzine	Antihistamines	1 0 1 0 0 0 0 1 0 0 1 0 1 0
24	Terfenadine	Antihistamines	0 0 0 0 1 0 0 0 0 0 0 0 0 0
25	Cetirizine	Antihistamines	0 0 1 0 0 0 0 0 0 0 1 0 0 0
26	Fexofenadine	Antihistamines	0 0 0 0 0 0 0 0 0 0 0 0 0 0
27	Mepivacaine	Local anesthetics	0 1 0 0 1 0 0 0 0 0 0 0 0 0
28	Propanocaine	Local anesthetics	0 1 0 1 0 0 0 0 0 0 0 0 0 0
29	Prilocaine	Local anesthetics	0 1 0 1 0 0 0 0 0 0 1 0 1 0
30	Bupivacaine	Local anesthetics	0 0 0 0 0 0 0 1 0 0 0 0 0 0
31	Aminoglutethimide	Antineoplastics	1 1 1 1 1 0 1 1 1 0 1 1 0 0
32	Bicalutamide	Antineoplastics	0 0 0 1 1 0 0 0 0 0 1 1 0 1
33	Pindolol	β-Blockers	1 1 0 1 0 0 1 0 0 1 0 0 1 0
34	Propranolol	β-Blockers	0 0 0 0 0 0 0 0 0 0 0 0 1 0
35	Metoprolol	β-Blockers	0 0 0 0 0 0 0 0 0 0 0 0 0 0
36	Acebutolol	β-Blockers	0 0 0 0 0 0 0 0 0 0 0 0 1 0
37	Atenolol	β-Blockers	0 0 0 0 0 0 0 0 0 0 0 0 0 0
38	Salbutamol	β-Blockers	0 0 0 0 0 0 0 0 0 0 0 0 0 0
39	Timolol	β-Blockers	0 0 0 0 0 0 0 0 0 0 0 0 0 0
40	Bambuterol	Bronchodilators	0 0 0 0 0 0 0 0 0 0 0 0 0 0
41	Isoprenaline	Bronchodilators	0 1 0 1 1 0 1 0 1 1 1 1 0 1
42	Orciprenaline	Bronchodilators	0 0 1 0 0 0 0 0 0 0 0 1 0 0
43	Clenbuterol	Bronchodilators	0 1 0 1 0 0 0 0 0 0 0 0 0 0
44	Terbutaline	Bronchodilators	0 0 0 0 1 0 0 0 0 0 0 0 0 0
45	Verapamil	Calcium channel blockers	0 0 0 0 0 0 0 0 0 0 0 0 0 0
46	Felodipine	Calcium channel blockers	0 1 0 0 0 0 0 1 0 0 0 0 0 0
47	Cinildipine	Calcium channel blockers	0 0 0 0 0 0 0 0 0 1 0 0 0 0
48	Procyclidine	Anticholinergics	0 0 0 0 0 0 0 0 0 0 0 0 0 0
49	Trimeprazine	Phenothiazines	0 0 0 0 0 0 0 1 0 0 0 0 0 0
50	Etopropazine	Phenothiazines	0 0 1 0 0 0 0 0 0 0 0 0 0 0
51	Promethazine	Phenothiazines	0 0 1 0 0 0 0 1 0 0 1 1 0 0
52	Thioridazine	Phenothiazines	0 0 1 0 0 0 0 0 0 0 0 0 0 0
53	Pantoprazole	Proton pump inhibitors	0 1 1 1 1 0 1 0 1 0 0 1 0 1
54	Methadone	Analgesics	0 0 1 0 0 0 0 1 0 0 0 0 0 0
55	Rabeprazole	Proton pump inhibitors	0 1 0 1 1 0 1 0 1 1 1 1 0 1
56	Lansoprazole	Proton pump inhibitors	0 1 0 1 1 0 1 0 1 0 1 1 1 1

C1, cellulose tris(3,5-dimethylphenylcarbamate); C2, cellulose tris(3-chloro-4-methylphenylcarbamate); C3, cellulose tris(4-methylbenzoate); C4, cellulose tris(4-chloro-3-methylphenylcarbamate); C5, immobilized cellulose tris(3,5-dichlorophenylcarbamate); A1, amylose tris(3,5-dimethylphenylcarbamate); A3, immobilized amylose tris(3-chloro-5-methylphenylcarbamate); a, acetonitrile; m, methanol

Compositions of the mobile phases

NH_4HCO_3 (5 mM, pH = 8.0)/ACN: 2/98; 5/95; 10/90; 20/80; 30/70; 40/60; 50/50; 60/40; 70/30; 80/20 (v/v)

NH_4HCO_3 (5 mM, pH = 8.0)/MeOH: 10/90; 20/80; 30/70; 40/60; 50/50; 60/40; 70/30 (v/v)

Table S2. Structural descriptors used for modelling.

Number	Symbol	Description
x_1	*C*X*	Number of heteroatoms bonded to the chiral carbon (C*-heteroatoms)
x_2	*C*XH*	Number of –OH or NHR groups bonded to the chiral carbon (C*-OH or C*-NHR)
x_3	*C*hA*	Number of aromatic heterocycles groups bonded to the chiral carbon (C*-aromatic heterocycles)
x_4	*C*a*	Number of aliphatic groups bonded to the chiral carbon (C*-aliphatic)
x_5	*C*H*	Number of hydrogen atoms bonded to the chiral carbon (C*-H)
x_6	*C*C=O*	Number of carbonyl groups bonded to the chiral carbon C*- amide (–C(=O)NR_1R_2), carbonyl (-C(=O)-R) and ester (–C(=O)-OR) groups
x_7	*C*A*	Number of aromatic groups bonded to the chiral carbon (C*-aromatic)
x_8	*Mr*	Molecular weight
x_9	*HBA*	**H-bond acceptors:** number of hydrogen bond acceptors in the molecule
x_{10}	*HBD*	**H-bond donors:** number of hydrogen bond donors in the molecule
x_{11}	*PSA*	Polar surface area
x_{12}	*ST*	Surface tension
x_{13}	*abc*	**Aliphatic bond count:** number of non-aromatic bonds in the molecule (excluding bonds of hydrogen atoms)
x_{14}	*Abc*	**Aromatic bond count**: number of aromatic bonds in the molecule
x_{15}	*bc*	**Bond count**: number of bonds in the molecule including hydrogens
x_{16}	*Rbc*	**Ring bond count**: number of ring bonds
x_{17}	*aRc*	**Aliphatic ring count**: number of those rings in the molecule, which have non-aromatic bonds
x_{18}	*ARc*	**Aromatic ring count**: number of aromatic rings in the molecule
x_{19}	*cRc*	**Carbo ring count**: number of those rings in the molecule, which contain carbon atoms only
x_{20}	*fARc*	**Fused aromatic ring count:** number of aromatic rings having common bonds with other rings
x_{21}	*fRc*	**Fused ring count**: number of fused rings in the molecule (having common bonds)
x_{22}	*HRc*	**Hetero ring count**: number of rings in the molecule, which contain hetero atoms
x_{23}	*HaRc*	**Heteroaliphatic ring count**: number of aliphatic heterocycles in the molecule
x_{24}	*HARc*	**Heteroaromatic ring count**: number of aromatic heterocycles in the molecule
x_{25}	*Rc*	**Ring count:** number of rings in the molecule
x_{26}	*RSc*	**Ring system count:** number of disjunct ring systems
x_{27}	*sRSs*	**Smallest ring system size:** number of rings in the smallest ring system
x_{28}	*Bi*	**Balaban index**: Balaban distance connectivity of the molecule, which is the average distance sum connectivity
x_{29}	*fsp3*	number of sp^3 hybridized carbons divided by the total carbon count
x_{30}	*C*	Number of carbon atoms in the molecule

Number	Symbol	Description
x_{31}	*O*	Number of oxygen atoms in the molecule
x_{32}	*S*	Number of sulfur atoms in the molecule
x_{33}	*Cl*	Number of chlorine atoms in the molecule
x_{34}	*F*	Number of fluor atoms in the molecule
x_{35}	*OH*	Number of –OH groups in the molecule
x_{36}	*NHR*	Number of -NHR groups in the molecule
x_{37}	*NR2*	Number of-NR_2 groups in the molecule
x_{38}	*ROR*	Number of R-O-R groups in the molecule
x_{39}	*tB*	Number of tert-butil groups in the molecule
x_{40}	*iP*	Number of iso-propyl groups in the molecule
x_{41}	*ACH3*	Number of -CH_3 groups bonded to an aromatic ring in the molecule
x_{42}	*ACl*	Number of Cl atoms bonded to an aromatic ring in the molecule
x_{43}	*AOH*	Number of -OH groups bonded to an aromatic ring in the molecule
x_{44}	*AOR*	Number of –OR groups bonded to an aromatic ring in the molecule
x_{45}	*ACOOR*	Number of -COOR groups bonded to an aromatic ring in the molecule
x_{46}	*ANHCOR*	Number of - NHCO-R groups bonded to an aromatic ring in the molecule
x_{47}	*ACA*	Number of moieties Ar-C-Ar
x_{48}	*A12*	Number of aromatic groups with 1,2 substitution.
x_{49}	*A14*	Number of aromatic groups with 1,4 substitution
x_{50}	*A123*	Number of aromatic groups with 1,2,3 substitution
x_{51}	*A124*	Number of aromatic groups with 1,2,4 substitution
x_{52}	*NA*	Number of nitrogen aromatic groups
x_{53}	*NRC*	Number of tertiary amines in aliphatic cycles
x_{54}	*SC*	Number of S atoms in aliphatic cycles
x_{55}	*logP*	Logarithm of the partition coefficient
x_{56}	*logD*	log *P* at working pH

Table S3. Customized criteria applied during CCLNNA optimization for evaluating "Success" and "Attempt". See further details in Experimental section

Case [a]	Recommendation [b]	**Yc**	IRS (**Y**)	Experimental (**T**)	Success	Attempt
1.1	R_1	≥ 0.5	1	1	1	1
1.2	R_2					2
1.3	R_3					3
2.1	R_1	≥ 0.5	1	0	-1	1
2.2	R_2					2
2.3	R_3					3
3.1	R_1	[0.25 < **Yc** < 0.5]	0	0	0.85	1
3.2	R_2					2
3.3	R_3					3
3.4				1 [c]	-0.95	3
4.1	R_1		1	1	0.8	1
4.2	R_2					2
4.3	R_3					3
5.1	R_1	≤ 0.25	0	0	0.9	0
5.2	R_2			1	-1	0
5.3	R_3			1 [c]	-0.95	0

[a] Case encompasses all possible scenarios for a given compound *i*.

[b] R_1, R_2 and R_3 refers to the first, second and third recommendation of CSP/MP system. Recommendations are used sequentially: 1st R_1; 2nd R_2 (if "Success" < 0 is obtained for R_1); 3rd R_3 (if "Success" < 0 is obtained for R_2).

[c] **T** = 1 corresponds to a CSP/MP system other than the ANN-recommendation.

Table S4. Exploration of the topic Intelligent recommendation system (IRS)

**Problem**	Consider analysts/researchers who need to enantioresolve a target compound without previous bibliographic information. They have access to a series of CSPs and MPs. Traditionally, they use a trial-and-error approach with different CSP/MP combinations, resulting in high costs, waste production, and time consumption.
**Human recommender?**	A hypothetic alternative, more in line with the SDG targets, would be to seek advice from recognized experts in chiral separations, able to recommend CSP/MP systems by examining the molecule's structure (if such human recommenders exist). Based on their experience and using the molecular structure of the target compound (or related data), the experts could recommend systems to be tested from the available CSP/MP options. A suitable assessment could be three hierarchically ranked CSP/MP recommendations (R_1, R_2, and R_3 to be tested in this order until successful). Furthermore, recommendations indicating that "none of your systems appear to enantioresolve the compound" are also valuable. However, the usefulness of such an alternative may be uncertain due to the inherent complexity of the task.
**Conventional Modelling**	Models optimized solely for predictive or classifying purposes may have limited recommendation capabilities. Previous results in our research group demonstrated that CCLNNA-ANNs optimized just to fit **T** have an unsuitable number of failures in their subsequent CSP/MP recommendations.
**Intelligent Recommendation system (IRS)**	An innovative alternative could be to replace the expert with a virtual counterpart (i.e., an IRS). This IRS would have to learn from the relationship between the molecular descriptors of a set of compounds and their corresponding experimental data on enantioresolution in different CSP/MP systems, and then become an expert on the modeled chromatographic systems. If the IRS learns effectively, it could offer the desired CSP/MP recommendations (e.g., R_1, R_2, and R_3) in hierarchical order or indicate when enantioresolution is impossible. Given that the task assigned to the IRS is complicated, it is logical to evaluate its potential with a limited set of available compounds and CSP/MP systems. The key to the IRS should be directly optimizing its recommending capability. This proof-of-concept could lead to further research for a more comprehensive CSP/MP-IRS. Neural network structures (CCLNNA, ANNs) appear to be suitable to handle the anticipated complexity. However, optimized-ANNs recommendations may differ, and CSP/MP recommendations could emerge from a collaborative strategy (i.e., a consensus model), which would increase decision certainty. Positive outcomes/results from this proof-of-concept would justify further research to develop a CSP/MP-IRS with broader applicability (e.g., a higher number of CSPs, MPs, and compounds), although this is beyond the scope of the current study.

Approximation to the relative importance of the molecular descriptors

There have been reported several approaches to approximate the relative importance of the input **X**-variables when utilizing an ANN. Unfortunately, there is no literature evidence regarding which approach could offer accurate estimations. The joint examination of various CCLNNA-ANN processes allows to calculate the frequency of the selected descriptors (i.e., those with remarked recommendation capability). This new indirect approximation enables determining the relative importance of the descriptors. Figure 1 illustrates the relative frequency (in percentage) of each molecular descriptor of the 11 ANNs shown in Table 1.

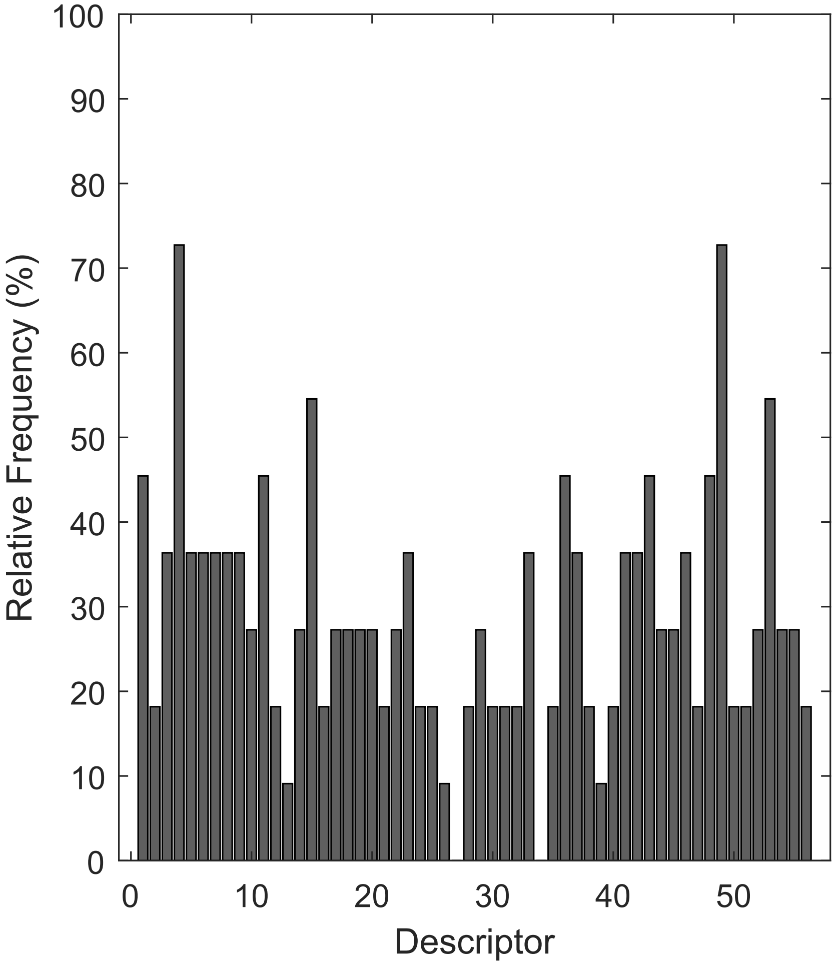

Figure S1. Relative frequency of each molecular descriptor of the 11 ANNs from Table 1.

The descriptors with the highest frequency include: $\mathbf{x}_4$ (C*a, number of aliphatic groups directly attached to the chiral atom) and $\mathbf{x}_{49}$ (number of aromatic groups with 1,4 substitution). Subsequently, descriptors $\mathbf{x}_{15}$ (number of bonds including hydrogen atoms) and $\mathbf{x}_{53}$ (number of tertiary amino groups in aliphatic cycles). Other descriptors have relative frequencies below 50%. It is important to note that, besides $\mathbf{x}_4$, the remaining C* parameters (the first seven descriptors), excluding descriptor $\mathbf{x}_2$, appear with certain frequency, suggesting their relevance in the enantioseparation processes. This suggests that in a future extended model (e.g., with more compounds), the C* parameters are likely to become more significant.
